# The experiences of night shift workers following three different dietary weight loss interventions: a qualitative study using behaviour change theory

**DOI:** 10.1186/s12966-025-01750-7

**Published:** 2025-05-28

**Authors:** Corinne Davis, Sue Kleve, Catherine E. Huggins, Maxine P. Bonham

**Affiliations:** 1 Department of Nutrition, Dietetics & Food, Faculty of Medicine, Nursing and Health Sciences, Monash University, Notting Hill, Australia; 2 Institute for Health Transformation, Global Centre for Preventive Health and Nutrition, School of Health and Social Development, Faculty of Health, Deakin University, Geelong, Australia

**Keywords:** Shift work, Qualitative study, Intermittent fasting, Diet, Nutrition, Work schedule, Obesity, Overweight

## Abstract

**Background:**

Shift workers are an estimated 15%—30% of the workforce in developed countries, who are disproportionally at risk of living with overweight or obesity. Dietary guidance is a component recommended for obesity management, however lacks consideration of the lifestyle and circadian disruption experienced by night shift workers. There is a lack of evidence addressing both weight loss and the metabolic consequences of eating at night. Intermittent fasting (IF) may provide metabolic benefits if fasting is aligned with night shifts. The Shifting Weight using Intermittent Fasting in night shift workers study compares three weight-loss interventions: 1) continuous energy restriction (CER); or twice-per-week IF with 2) fasting during night shifts or 3) day fasting. This study aims to explore the experiences of participants while following the interventions to understand how intervention features and external enablers or barriers influence engagement.

**Methods:**

Forty-seven semi-structured interviews (22 baseline, 25 follow-up) were conducted with 33 participants. Eighteen participants also completed optional fortnightly audio diaries to enrich data collected on experiences over time. Interviews and diaries were analysed using the five-steps of framework analysis and themes were deductively mapped to behaviour change frameworks and the social-ecological model.

**Results:**

Analysis resulted in seven major themes and 27 subthemes. Three main themes describe intervention factors influencing engagement: 1) Simplicity and ease, 2) Support and accountability, and 3) An individualised approach is sometimes needed. Four themes described external factors to the interventions influencing engagement: 4) Personal motivation and attitudes, 5) Physiological influences of eating behaviours, 6) Social support at home and work, and 7) Work structure and environment.

**Conclusions:**

Across all three interventions, participants valued the ease of interventions, which was the clear dietary prescription and focus on two days per week for IF, or a focus on small changes for CER. Behavioural regulation and providing meals/snacks were identified as critical features. Modifications to address identified enablers/barriers include: providing flexible fasting periods; addition of fatigue management initiatives; increased focus on non-weight related health changes during periods of slowed weight-loss; implementation in workplace settings to harness social support; and providing a healthier food environment.

**Supplementary Information:**

The online version contains supplementary material available at 10.1186/s12966-025-01750-7.

## Background

An estimated 15%—30% of the workforce in developed countries undertake shift work, which often involves working at night in critical fields such as healthcare, emergency services, transport, security and manufacturing [1]. Working night shifts is associated with a greater risk of living with overweight or obesity, and obesity related conditions such as type 2 diabetes, cardiovascular disease, and several types of cancer when compared to day working counterparts [2–5]. While the link between night shift work and living with overweight or obesity is complex and likely multifactorial, evidence supports that night-time eating is linked with impaired glucose tolerance and insulin resistance which is compounded by poorer food choices [6]. While current guidelines for obesity management recommend a multidisciplinary approach, reducing daily energy intake is a core component [7–10]. Implementing strategies to reduce energy intake is challenging for night shift workers due to multiple barriers: at the intrapersonal level (i.e., characteristics of the individual), such as fatigue, time constraints, disruption to the alignment of eating patterns with circadian rhythms; interpersonal level (i.e., social network and support systems), such as the influence of workplace social norms around eating at night; and at the organisational or community level (i.e., workplace settings and the relationship between organisations), such as limited availability of healthy food within the workplace and community at night [11–14]. More recent research in support of these findings has used behaviour change theory to explore the influences of dietary behaviour for night shift workers, identifying meal planning templates, self-monitoring and biofeedback, and increasing access to healthy food options may help address these influences [15]. However, there is a dearth of quantitative and qualitative research that has explored not only the effectiveness, but also the enablers and barriers influencing engaging with dietary weight-loss interventions for night shift workers [11].

Two dietary lifestyle intervention studies, designed specifically for night-shift workers, provide some qualitative evidence for the factors influencing engaging with a weight-loss intervention [16, 17]. Huggins et al*.* [16] explored the feasibility of a meal timing intervention aimed at avoiding dietary intake between 01:00 and 06:00 during the night shift. Individual motivation was important, with the perceived health benefits of the intervention related to weight loss a key motivator [16]. While the absolute avoidance of dietary energy intake during the specified hours of the night shift was achievable, it was challenging given the typical use of food and drinks (especially caffeine) to deal with the burden of shift work and fatigue [16]. Additionally, the workplace social and physical environment presented barriers, such as being surrounded by colleagues consuming food during breaks [16]. A more recent qualitative study by Sooriyaarachchi et al*.* [17], explored the experiences of night shift workers using a low-calorie/energy meal replacement for weight-loss. Participants expressed satisfaction towards the intervention, with its perceived simplicity and convenience making it feasible even during busy night shifts [17]. Strong determination and motivation, experiencing weight-loss and other associated health benefits supported adherence to the intervention [17]. While these existing studies [16, 17] provide some important considerations for what drives night-shift working participants to engage with behaviours that promote weight-loss, both interventions were for short durations (i.e., four or eight weeks), only one intervention had a primary outcome of weight loss [17], and qualitative data collection was limited to post-interventions interviews, potentially leading to recall bias.

The Shifting Weight using Intermittent Fasting in night shift workers (SWIFt) study is a trial that compares three dietary weight-loss interventions [18]. In summary, SWIFt tests the effectiveness of a modified intermittent fasting dietary regimen (referred to as IF hereon) that involves habitual dietary intake on five days and limited energy consumption (20%—25% of energy requirements) on two ‘fast’ days per week [18]. Studies in non-shift workers have shown that weight loss with IF is similar to continuous energy restriction (CER) [19], with fasting at night potentially providing greater metabolic benefits due to a reduction in overnight energy consumption [18]. Furthermore, for night shift workers who often experience fatigue, inconsistent routines and resulting lack of motivation for healthier dietary practices [11], IF may be a flexible approach due to limiting dietary manipulation to only two days per week [18]. The SWIFt study offers a unique opportunity to explore the experiences of night-shift workers while following a weight-loss intervention to determine the factors that support or hinder this endeavour. This exploration is essential to inform scale up [20, 21] of the interventions and for designing and implementing future interventions tailored to night-shift workers. The aim of this qualitative study was to explore the experiences of participants during the 24-week intervention period to understand:

- How intervention features influence engagement, and
- How enablers and barriers at the individual, social, organisational, and community levels (external factors), influence engagement.

## Methods

This qualitative study forms part of a wider mixed methods study that was employed to evaluate the SWIFt randomised controlled trial [22, 23]. This study is centred in ‘pragmatism’ as a theoretical paradigm guiding research design, which is oriented to choosing multiple, best-fit methods to answer the research questions posed to gain theory that informs effective practice that can be immediately applied to real world settings [23–25]. In this sense, this qualitative study focuses on “how” the intervention is perceived to operate for participants within their context based on exploring their experiences in order to help inform future implementation of dietary interventions involving night shift workers.

### SWIFt study design, participants and intervention

**Fig. 1:**
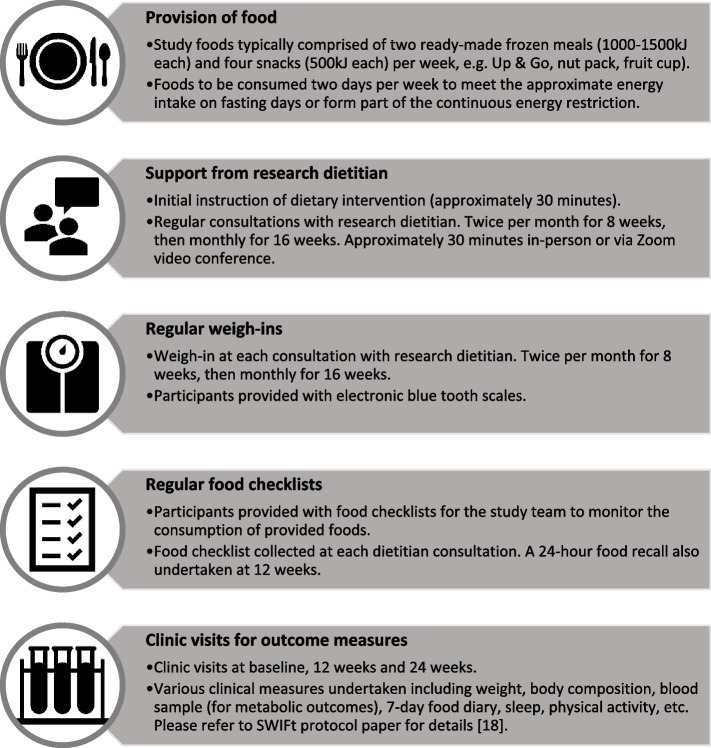
Intervention features of the Shifting Weight using Intermittent Fasting in night shift workers (SWIFt) study

The SWIFt study is a randomised controlled dietary weight loss trial with a 24-week intervention period. The details of the interventions have been previously published [18, 22]. The study compared three dietary intervention groups. Two groups undertook an IF dietary regimen undertaking two fast days per week (energy intake restricted to 2100 – 2500 kJ/day), with one group fasting during night shifts (IF:2N) and one group fasting on day shifts or days off (IF:2D). Habitual dietary intake was the aim of the remaining five days of the week. The two IF groups were compared with an active control group that undertook continuous energy restriction (CER), aiming for a daily 20% energy restriction guided by the Australian Dietary Guidelines (ADG) and the Australian Guide to Healthy Eating (AGHE) [26]. Figure 1 details the intervention features of the SWIFt study, which included provision of some food, support from a research dietitian, regular weigh-ins, completion of food checklists, and clinic visits for outcome measures.

Eligibility criteria and recruitment for the overall SWIFt study has been previously described [18]. In summary, study participants were eligible to participate if they were aged between 25 and 65 years, worked a minimum of two nights per week (a minimum of six hours between 2000 h – 0600 h including travel time) for at least six consecutive months, had a body mass index (BMI) of ≥ 28 kg/m^2^ for non-Asian men or women and ≥ 26 kg/m^2^ for Asian men or women, and were able to attend the respective study site in Melbourne or Adelaide, Australia [18]. BMI was measured at a screening visit to confirm eligibility. The previously published study protocol outlines exclusion criteria relating to medical conditions or medication that may affect body composition and metabolism (e.g., participants diagnosed with a medical condition such as diabetes, cardiovascular disease or inflammatory bowel disease, or requiring medications such as antihypertensive, antidepressants, thyroxine, glucocorticoids) [18]. Other exclusion criteria included being pregnant, planning a pregnancy, breastfeeding, previous weight-loss surgery, and dietary restrictions (e.g., allergies) or extended work leave that effects the ability to follow the dietary protocol [18].

### Participant recruitment

Participants who consented to be involved in the qualitative study were contacted via email after randomisation to one of the SWIFt interventions. Participants were invited to participate in the qualitative component based on a maximum-variation sampling approach and were invited to participate in interviews with an option to also complete audio diaries [27]. Maximum variation sampling was used to provide a diverse set of participant viewpoints across age, gender, occupation, shift-type, intervention group and study completion. Preliminary data analysis occurred concurrently, and recruitment ceased once maximum-variation was met and when no new themes were identified.

### Data collection

Self-reported demographic and employment details and researcher measured body weight details were collected from participants at baseline for the overall SWIFt study, as previously described [18]. Qualitative data were collected and reported in line with the consolidated criteria for reporting qualitative research (COREQ) [28].

#### Semi-structured interviews

In-depth, semi-structured interviews were undertaken by CD, SK and GL with participants at baseline and/or 24 weeks. An interview guide for each stage of interviews was informed by existing dietary research in the shift working population [29, 30] and the Theoretical Domains Framework (TDF) (Additional file 1). The TDF provides a comprehensive set of constructs that have been derived from a review of relevant behaviour change theories [31]. It has been successfully used for analysing interview data in a shift worker population regarding dietary choices [29]. Interviews were completed over the phone or via Zoom video-conference (Zoom Video Communications Inc., Version 5.13) at a time convenient to the study participant. Participants were requested to choose a quiet, personal space for their interview. Before interview commencement, the interviewer introduced themselves and their role in the study and provided background to the purpose of the qualitative study (Additional file 1). Researcher reflexivity is an important component to allow for an awareness of how a researcher’s positionality may influence the research process, and to provide transparent and high-quality qualitative research [32, 33]. Researcher CD conducted the majority of interviews and had no prior relationship with study participants. She is an accredited practising dietitian undertaking this work as part of her PhD studies, with prior experience in qualitative research, and was involved in the wider SWIFt study as a researcher collecting data and as a research dietitian undertaking dietary consults with SWIFt participants. Interviews with participants assigned to CD as a dietitian (*n* = 2) were undertaken by another member of the research team (SK and GL) to allow participants to more freely discuss their experience. The interviews were audio recorded and transcribed verbatim by researcher CD or a transcription service and checked by researcher CD.

#### Longitudinal audio diaries (LADs)

A subset of participants provided an approximate five-minute, fortnightly longitudinal audio diary (LAD) account of their dietary intervention experience over the 24-week period. LADs are a unique, flexible and useful tool for enriching qualitative data collected on experiences over time (i.e., do not rely on memory recall), where participants may more freely disclose matters of personal salience without the presence of a researcher [34]. Participants were encouraged to use their mobile/smart phone to audio record their diary. A LAD prompt sheet was provided to participants (Additional file 2) to provide questions for participants to consider when recording their audio diary, which was designed based on previous research using LADs as an evaluation tool [35]. The LADs were listened to by researcher CD within one working day. After listening to the LAD, if issues of concern were raised, such as participant distress, this was discussed with the wider research team for appropriate next steps (e.g., reminder of support services available as outlined in the SWIFt study explanatory statement). The LADs were transcribed verbatim by the main researcher (CD) or a transcription service and checked by researcher CD.

### Data analysis

Interview and LAD transcripts were entered into NVivo (Lumivero, Version 12) and analysed using the five-steps of ‘framework analysis’ [36, 37]. In step one (familiarisation), a subset (*n* = 6) of transcripts representing a mix of dietary intervention groups were analysed inductively by researcher CD and reviewed by researcher SK to identify influencing factors (intervention features in addition to enablers and barriers) for participant engagement. Engaging with the intervention was considered to cover a broad array of aspects, including adhering to the requirements of the dietary interventions, attending dietetic reviews, attending clinic visits, completing measures, and remaining in the study (i.e., not withdrawing). Step two (identifying a thematic framework) involved developing an initial coding framework based on step one. In step three (indexing), the framework was used to code all data in NVivo by researcher CD with a subset cross-checked by researcher SK initially in the early stages of coding (*n* = 6) and the later stages (six interviews and three LAD sets). CD and SK met at each step to discuss feedback and agreement was achieved through discussion, negating the need of a third researcher. During analysis, audio files were listened to alongside reading the transcript. Each coded text for intervention enablers was deductively mapped to the following behaviour change frameworks: the COM-B model [38], the Theoretical Domains Framework (TDF) [31], and the Behaviour Change Taxonomy (BCT) [39], which are often used to evaluate behaviour change interventions [38]. The mapping process explored the behaviour change mechanisms related to the intervention features in line with recommendations for identifying active ingredients of interventions to aid translation of interventions into practice settings [40]. Enablers and barriers external to the intervention were deductively mapped to the domains of the social-ecological model (SEM) [14] to guide developing themes and subthemes. The SEM consists of five distinct domains, including intrapersonal (i.e., individual), interpersonal (i.e., social), organisational, community, and public policy [14]. The SEM considers how these factors, inclusive of and beyond the individual sphere, interact to influence health behaviours, enabling the development of strategies to improve behaviours [14]. In step four (charting), any differences between the dietary interventions and participant characteristics were identified. Finally, step five (mapping and interpretation) involved interpreting the findings based on existing literature which is provided within the discussion section of this paper.

Reflexive diaries were completed by the main researcher at key data points along the research process, including: after participant interviews, after reviewing LADs, and after each data analysis step. Insights into this process were discussed, as appropriate, at fortnightly meetings with the wider researcher team.

## Results

Forty-seven interviews were collected from 33 participants out of the 250 participants randomised to the SWIFt study. Twenty-two baseline interviews, 20 twenty-four-week interviews, and five withdrawal or loss-to-follow up interviews were completed. Table 1 provides detail of the number of participants who completed various forms of interviews and audio diaries. Twenty-one participants declined or were non-responsive for an invitation for an interview during active participation in the wider study and 18 participants were non-responsive for an invitation for a withdrawal or loss-to-follow up interview. No differences in age, gender, employment and body weight variables were observed between participants who opted in to the qualitative study compared with participants who were non-responsive/declined to participate. However, there was a higher percentage of participants who identified within the European or Australian cultural and ethnic groups who opted to complete interviews or audio diaries (97%) compared with participants who were non-responsive/declined (65%). The majority of interviews (*n* = 30; 64%) were completed over the phone, with the remainder via Zoom video-conference. Each interview ranged from 26 to 70 min (average = 45). Eighteen out of the 33 participants also completed audio-diaries (minimum = one diary; maximum = 12 diaries; mean = eight diaries). Each individual audio-diary ranged from 35 s to 18 min (average = four minutes). Interviews and audio-diaries generated 43 h of data. There was a broad mix of participants across demographic, employment and dietary intervention variables and characteristics were consistent with the wider SWIFt study (Table 2).

**Table 1:** Number of participants who completed various qualitative forms of data collection for the SWIFt study

Qualitative data type	Number of participants
Baseline interview^a^	2
Baseline interview + audio diaries^b^	6
Baseline interview + 24-week interview	1
Baseline interview + audio diaries + 24-week interview	13
24-week/withdrawal/lost-to-follow up interview^c^	11

*Abbreviations*: *SWIFt* Shifting Weight using Intermittent Fasting in night shift workers

Symbols ^a^One participant was non-responsive to request for 24-week interview and one participant formally withdrew from the study

^b^Three participants formally withdrew from the study and three participants were non-responsive to request for 24-week interview

^c^Six participants were already active in the SWIFt study once the qualitative component of the study commenced and only completed 24-week interviews

**Table 2:** Baseline characteristics of participants in the qualitative component of the SWIFt study

Demographics	(*n* = 33)
Age (years), mean$\pm$SD	45.2$\pm$9.7
Gender, n (%)	
Female	18 (54.5)
Male	15 (45.5)
Cultural and ethnic group, n (%)^a^	
European^b^	28 (84.8)
Asian^c^	1 (3.0)
Australian^d^	4 (12.1)
Other^e^	0
**Employment**	
Occupation, n (%)	
Managers	0
Professionals	10 (30.3)
Community and Personal Service Workers	5 (15.2)
Clerical and Administrative Workers	5 (15.2)
Labourers	5 (15.2)
Machinery Operators and Drivers	5 (15.2)
Technicians and Trades	3 (9.1)
Shift schedule, n (%)	
Permanent/fixed night shifts	19 (57.6)
Rotational shifts	14 (42.4)
Split shift	0
Years in shift work, mean$\pm$SD	13.8$\pm$7.9
**Dietary intervention allocation**	
Continuous energy restriction (CER), n (%)	10 (30.3)
Intermittent fasting during day (IF:2D), n (%)	9 (27.3)
Intermittent fasting during night shift (IF:2N), n (%)	14 (42.4)
**Body weight at baseline**	
Body weight (kg), mean$\pm$SD	104.5$\pm$17.0
Body mass index (kg/m^2^), mean$\pm$SD	36.0$\pm$5.7

*Abbreviations*: *SWIFt* Shifting Weight using Intermittent Fasting in night shift workers

Symbols: ^a^The Australian Standard Classification of Cultural and Ethnic Groups was used [41]

^b^Includes North-west European (e.g. British, Irish, Dutch, German) and Southern and Eastern Europe (e.g. Italian, Spanish, Greek, Macedonian)

^c^Includes Southern and Central Asian (e.g. Indian, Pakistani) and South East Asian (e.g. Vietnamese, Indonesian), and North East Asian (e.g. Chinese, Japanese, South Korean)

^d^Inlcudes participants who specified ‘other’ and indicated ‘Australian’

^e^Includes People of the Pacific Islands (e.g. Maori, Samoan), People of the Americas (i.e. North American, South American), Sub-Saharan African, North African and Middle Eastern (e.g. Arab, Jewish), not stated or multiple ethnicities, New Zealander

From the analysis of participants’ experience in following their allocated dietary intervention, seven major themes and 27 associated sub-themes were identified and summarised in the following section. The first three themes relate to SWIFt intervention factors influencing engagement and the remaining four themes relate to external factors to the SWIFt interventions. The following section also presents illustrative LAD quotes that highlight experiences over time as applicable. More commonality than differences between intervention groups was identified, therefore results have been presented by theme rather than by intervention group. The main differences between intervention groups were: different intervention components between groups were perceived to provide simplicity to the approach; building nutrition knowledge and a focus on regular eating was particularly important for dietetic support for the CER intervention; and a limited focus on non-fasting periods impacted engagement for some participants following the IF interventions. The barriers of fatigue and a lack of time were more commonly reported by participants following the CER intervention compared with the IF interventions. These differences are discussed in detail within the following description of themes and subthemes. To further highlight the differences identified between intervention groups, Table 3 presents all themes, subthemes, illustrative quotes, whether a subtheme was identified as an enabler or barrier, and which dietary intervention the subtheme was more commonly reported.

**Table 3:** Summary of themes, subthemes and illustrative quotes identified through thematic framework analysis of interviews and longitudinal audio diaries (LADs) for the SWIFt study

Themes and subthemes	Illustrative quotes	CER	IF:2D	IF:2N
**SWIFt intervention factors influencing engagement**				
***Theme 1: Simplicity and ease***				
1.1 Ease and acceptability of provided foods ( +)	*…I'm not someone who's well organised with food for night shift…*[it] *was really easy and convenient to be on the low nights* [fasting night] *and…all sort of prepped and ready to go.* (Interview with participant 2062; Female; IF:2N)	✓	✓	✓
1.2 Straight forward and flexible approach ( +)	*…doing the 5 and 2, I don't even have to think about the fasting days because this is what I'm having. And then on the non-fasting days, yeah. Just have whatever I feel like.* (Interview with participant 1084; Female; IF:2D)		✓	✓
1.3 Inflexibility of fasting periods (-)	*But the fact that you're limited to the numbers of days that you can do the diet, I found it was very hard. And the fact that it is the days off, you know, a lot of people like to go out and be social.* (Interview with participant 1055; Male; IF:2D; works 4 – 5 night-shifts per week) *I didn't do any back-to-back nights this fortnight, which made a huge difference and was much easier. I was much less ‘hangry’, and yeah, it was just quite manageable.* (LAD from participant 2062; Female; IF:2N; rotational shift schedule with predominantly consecutive night shifts)		✓	✓
1.4 Fasting side effects (-)	*I was just getting stomach pain from being so hungry. Yeah, I ended up getting headaches. And then I was making mistakes at work…just got harder and harder. And the negatives were outweighing the positives.* (Interview with participant 1123; Male; IF:2N)		✓	✓
1.5 Easier with time ( +)	*It probably took about, I don’t know, three, four weeks, and then, yeah, I found it* [dietary intervention] *a lot easier.* (Interview with participant 1039; Female; IF:2D)		✓	✓
1.6 Small changes ( +)	*I haven't really found the diet too difficult. I'm still eating what I normally would just changing a few things of the ingredients to less fat ingredients. And if I do go out, I either don't eat as much of the item or I choose a different meal and the same at home – more portion size.* (LAD from participant 2052; Female; CER)	✓		
1.7 Burden of data collection for the wider SWIFt study (-)	*And it's not just a food diary. It's like…this is the degree in which we need you to fill it out. And there's, there's blood samples, stool samples, there's DEXA scans…it's a little bit overwhelming.* (Interview with participant 1070; Male; IF:2N)	✓	✓	✓
***Theme 2: Support and accountability***				
2.1 Dietetic support ( +)	*Then if you did have your down days, she* [research dietitian] *was always there to say, “You’re still losing weight. You haven’t gone backwards. It is healthier. You’re doing well.” It’s like your own cheer squad isn’t it…* (Interview with participant 2052; Female; CER)	✓	✓	✓
2.2 Accountability ( +)	*I guess I'm accountable to somebody, and it's keeping me honest. It's keeping me motivated that I've gotta answer to somebody…in the past, when it's just me, I can lie to myself.* (LAD from participant 1062; Male; IF:2N)	✓	✓	✓
2.3 Self-monitoring ( ±)	*…regularly seeing the weight on the scales actually going down for once was quite nice. That was a good motivation to stick to it.* (Interview with participant 1045; Male; IF:2N) *…few days where I've sort of haven't quite stuck to my window* [fasting period]. *And I think, it's been from lack of…results that you sort of get that, "Oh, well, this is not much point in weight loss terms."* (Interview with participant 2062; Female; IF:2N)	✓	✓	✓
***Theme 3: An individualised approach is sometimes needed***				
3.1 Increased nutrition knowledge ( +)	*I’ve learnt more about different foods and how one brand can be completely different to another brand. Whereas before, it used to be, “Oh whatever brand, doesn't matter if they're all the same”.* (Interview with participant 1038; Female; CER)	✓		
3.2 Focus on regular eating ( +)	*Because normally I would just have the breakfast and then not eat anything until either I picked the boys up, and then I’m rushing around, trying to get something for dinner.* (Interview with participant 2052; Female; CER)	✓		
3.3 Limited focus on non-fasting periods (-)	*…although the low days themselves are effective…the other four days at home, are probably much more detrimental overall to my week’s intake.* (Interview with participant 2062; Female; IF:2N)		✓	✓
**External factors to the SWIFt interventions influencing engagement**				
***Theme 4: Personal motivation and attitudes (intrapersonal level of SEM)***				
4.1 Motivation ( ±)	*I'd say initially, for the first two months, I did have a lot of motivation. But that waned in the last four to five weeks.* (Interview with participant 1123; Male; IF:2N)	✓	✓	✓
4.2 Positive mindset ( +)	*The scales weren’t being friends for a while, but then they would, and then they wouldn’t.* [Laughter] *But I just learned to not worry about it and just keep going. It’s only a number, it’s how you sort of feel on the inside.* (Interview with participant 2052; Female; CER)	✓	✓	✓
4.3 Lack of time (-)	*So, I don’t know why I thought that this was gonna be easy. It’s actually quite hard. We have a really busy lifestyle… we picked the bad options…but we really need to start thinking about other food.* (LAD from participant 2054; Female; CER)	✓**↑**	✓	✓
***Theme 5: Physiological influences of eating behaviours (intrapersonal level of SEM)***				
5.1 Fatigue (-)	*…lack of sleep affects your food choices. There was probably a part of me that was going, yeah, I would much rather have nachos than veggie green curry* [frozen meal for the fasting period]. (Interview with participant 1044; Female; IF:2D)	✓**↑**	✓	✓
5.2 Emotional eating (-)	*I need to try and find a strategy to help deal with the mental health especially for the fasting days. Because when I'm feeling worthless, I don't, fasting or doing anything positive for myself…* (LAD from participant 1084; Female; IF:2D*)*	✓	✓	✓
5.3 Keeping busy ( +)	*I need to make myself a bit busy or do something that I’m not gonna be sitting idle…go out for a little walk or something.* (Interview with participant 1040; Female; IF:2N)	✓	✓	✓
5.4 Mindful eating practices ( +)	*Nurses are notorious for eating fast because you only get half an hour. You’ve gotta eat fast to get everything done but, yeah, I purposely slowed down my eating so that meals took a lot longer.* (Interview with participant 1039; Female; IF:2D)	✓	✓	✓
***Theme 6: Social support at home and work (interpersonal level of SEM)***				
6.1 Supportive family, friends or colleagues ( +)	*…my family were all just eating the same stuff.* (Interview with participant 1031; Female; CER) *…it was nice to have people* [work colleagues] *to be like, "Cool. I won't offer you any* [biscuits] *tonight."* (Interview with participant 2062; Female; IF:2N)	✓	✓	✓
6.2 What others are eating (-)	*…it was easier to do it while I was a way from everyone else. When I came home, it was a lot harder, like, they'd be having, like, chicken parmies and I am having a rice, you know* [provided frozen meal]. (Interview with participant 2097; Male; IF:2D)	✓	✓	✓
***Theme 7: Work structure and environment (organisational/community levels of SEM)***				
7.1 Busy work ( ±)	*A godsend was I actually* [was] *quite busy at work. So I didn't have time to think about boredom eating…* (LAD from participant 2052; Female; CER)	✓**↑-**	✓	✓**↑-**
7.2 Regular work breaks ( +)	*But as a rule, I take everything* [meals and snacks] *in* [to work] *with me, and I monitor my meals. And I actually highly organise the shift around my diet*…(Interview with participant 2078; Male; CER)	✓	✓	✓
7.3 Shift schedule (-)	*I find* [with] *night shifts that I always have a real struggle actually to stick to anything. When I’m tired, I tend to look for food.* (Interview with participant 1041; Female; CER)	✓	✓	✓
7.4 Availability of unhealthy food (-)	*The difficulty with following the diet, was that over the last two weeks, they offered free* [laughter] *food at work* [chocolates, hot dogs, etc.]. (LAD with participant 2065; Female; IF:2N)	✓**↑**	✓	✓**↑**
7.5 COVID-19 stressors (-)	*…the more* [COVID] *lockdown we had the sort of more down I became…I was snacking more.* (Interview with participant 1044; Female; IF:2N)	✓	✓	✓

*Abbreviations*: *SWIFt* Shifting Weight using Intermittent Fasting in night shift workers, *CER* continuous energy restriction intervention, *IF:2D* intermittent fasting intervention with day fasting, *IF:2N* intermittent fasting intervention with night shift fasting, *SEM* social ecological model, *LAD* longitudinal audio diary

Symbols: + subtheme identified as an enabler,—subtheme identified as a barrier

✓ indicates that the subtheme was identified for the corresponding dietary intervention

**↑** subtheme more commonly reported in corresponding dietary intervention

Table 4 displays the mapping of the intervention enabler sub-themes, to behaviour change frameworks and outlines the frequency of coding to each domain. The enabler sub-themes were predominantly related to the ‘motivation’ and ‘capability’ domains of the COM-B model [38] and one sub-theme related to the ‘opportunity’ domain. Mapping to the ‘capability’ COM-B domain was more frequent for the CER intervention compared to the IF interventions. For the Theoretical Domains Framework (TDF) [31], the following domains were the most frequently reported: ‘behavioural regulation’, ‘knowledge’, ‘goals’ and ‘environmental context and resources’. For the Behaviour Change Taxonomy (BCT) [39], the following domains were the most frequently reported: ‘instruction on how to perform a behaviour’, ‘goal setting (behaviour)’, ‘self-monitoring of outcome(s) of behaviour’, and ‘adding objects to the environment’.

**Table 4:** Mapping of SWIFt study intervention enabler sub-themes to behaviour change frameworks

COM-B	TDF Domain	BCT	Enabler sub-theme
M [443]	Goals [152]	Goal setting (behaviour) [152]	1.2 Straight forward and flexible approach
			1.6 Small changes
			3.2 Focus on regular eating
			2.1 Dietetic support
			2.2 Accountability
	Behavioural regulation [225]	Self-monitoring of behaviour [20]	2.3 Self-monitoring
		Self-monitoring of outcome(s) of behaviour [112]	2.3 Self-monitoring
		Monitoring of behaviour by others without feedback [47]	2.2 Accountability
		Monitoring outcome(s) of behaviour by others without feedback [47]	2.2 Accountability
		Feedback on behaviour [7]	2.1 Dietetic support
		Feedback on outcome(s) of behaviour [14]	2.1 Dietetic support
		Graded tasks [20]	1.6 Small changes
	Intentions [64]	Commitment [64]	2.2 Accountability
	Social influences [22]	Social support (emotional) [22]	2.1 Dietetic support
	Memory, attention and decision processes [43]	Conserving mental resources [43]	1.1 Ease and acceptability of provided foods
			1.2 Straight forward and flexible approach
	None identified	None identified	1.5 Easier with time
C [163]	Knowledge [161]	Instruction on how to perform a behaviour [161]	1.1 Ease and acceptability of provided foods
			1.6 Small changes
			3.1 Increased nutrition knowledge
			3.2 Focus on regular eating
			1.2 Straight forward and flexible approach
			2.1 Dietetic support
	Beliefs about capability [5]	Verbal persuasion about capability [5]	2.1 Dietetic support
O (102)	Environmental context and resources [102]	Adding objects to the environment [102]	1.1 Ease and acceptability of provided foods

*Abbreviations*: *SWIFt* Shifting Weight using Intermittent Fasting in night shift workers, *COM-B* the Capability (C) Opportunity (O) Motivation (M) behaviour change model, *TDF *Theoretical Domains Framework, *BCT* Behaviour Change Taxonomy, *M* Motivation, *C* Capability, *O* Opportunity

Symbols: [n], frequency of coding for the behaviour change framework domain – note: some text was coded to multiple themes

### Theme 1: Simplicity and ease

This main theme relates to intervention components mostly described by participants as contributing to the simplicity and ease of their allocated intervention: the clear structure and focus on only two days per week for the IF interventions, and the small dietary changes for the CER intervention, as described at subthemes 1.2 and 1.6 below. Across all interventions, the provision of food also made the interventions easier to follow (subtheme 1.1). However, as described within subthemes 1.3 and 1.4, for some participants allocated to the IF interventions, the inflexibility of being randomised to either a night shift or day fasting period, and fasting side effects, made the approach less easy to follow. In addition, across all interventions, the burden of data collection negatively impacted on the ease of engaging with the study for a small number of participants (subtheme 1.7).

#### 1.1 Ease and acceptability of provided foods

The foods provided to participants as part of the study were mostly perceived as quick, easy to prepare, and palatable options. The foods discouraged participants from purchasing takeaway (fast foods) or unhealthy convenience foods for two days of the week, especially during the night shift for the IF:2N and CER interventions. The provision of foods on the fasting days for IF interventions removed the need for participants to plan or prepare foods, making it easier to keep within their energy restriction limits.

#### 1.2 Straight forward and flexible approach to weight loss

Participants often discussed that the IF interventions were clear, structured and there was no ambiguity of what was required. Focussing on only two days per week rather than every day also made the approach straight forward. For most participants, the two fasting days could be arranged to suit their lifestyle making it a flexible approach. For the IF:2N intervention, some participants found that the prescription of aligning fasting with a night shift provided a flexible approach that had less impact on social and family activities. Given that the approach was straight forward and flexible, sometimes participants described being motivated to improve their dietary intake on non-fasting days.

#### 1.3 Inflexibility of fasting periods

Fasting interventions did not always suit lifestyle or work schedule, due to the inflexibility of the prescribed fasting periods and varied work schedules. The IF:2D intervention was more challenging when fasting coincided with days off from work and clashed with social activities, which was more likely for participants working four to five-night shifts per week. Consecutive nights of fasting on night shift (often required for rotational shift workers) was also challenging and increased the chances of experiencing fasting side effects (discussed within subtheme 1.4). Some participants also expressed that the inflexibility of the fasting requirements (e.g., not adding milk to oatmeal or coffee) did not meet their preferences.

#### 1.4 Fasting side effects

While the fasting approaches were often reported as straight forward to follow, most participants reported side effects, which included hunger, headaches, lack of concentration, fatigue, nausea, moodiness, and interrupted sleep. Participants often incorporated strategies suggested by the research dietitian to mitigate the side effects, such as consuming extra permitted foods/drinks (e.g., low-calorie/energy vegetables and sugar free drink options were permitted) and increasing water intake. Participants also reported keeping busy, aiming for a break in between fasting days and distributing their provided food evenly over their fasting period.

*Pretty much straightforward* [following the dietary strategy]*…Obviously, you have your days where…it's harder, you got things to do…sometimes I have to go shopping, grocery shopping during my fasting day. The temptation to go get something. And your stomach rumbling.* (Interview with participant 1082; Male; IF:2D)

#### 1.5 Easier with time

The majority of participants reported that the fasting became easier with time. Analysis of the LADs revealed that most participants found the first two to four weeks the most challenging as their bodies adjusted to the fasting periods and were surprised at their ability to follow the approach as time progressed.

*The fasting process* [is] *actually getting quite much- it's easier* [then] *when I started and what I ever thought it would be.* (LAD from participant 1055 at Week 6; Male; IF:2D)

#### 1.6 Small changes

Participants often reported that making small changes to their existing diet made the CER intervention simple to follow. This also suited their lifestyle, particularly for participants who lived with their family. The focus on making small changes was also noted as particularly beneficial for night shifts. However, there were a small number of participants who identified that they would have liked more structure, such as a detailed and tailored meal plan for each day of the week, rather than suggested modifications to their current diet.

#### 1.7 Burden of data collection for the wider SWIFt study

The inflexibility of study visits for collecting data (e.g., the requirement to not be directly after a night shift and preferably in the morning for blood collection) was raised by a small number of participants (two participants that were lost to follow up and four participants raised this as a potential issue for other shift workers). Additionally, the number of measures to be completed as part of the study requirements added a burden to some participants that negatively impacted motivation to engage with the study overall.

### Theme 2: Support and accountability

Across all interventions, the support and accountability provided by the research dietitian was often mentioned as important for continued engagement with the dietary interventions (subthemes 2.1 and 2.2). A key difference between the interventions was that building knowledge was particularly important for dietetic support for the CER intervention. Across all interventions, self-monitoring of weight could be an enabler or barrier to individual accountability depending on their weight change experience (subtheme 2.3).

#### 2.1 Dietetic support

The aspects of support from the research dietitian that were described as enabling, included encouraging a more positive mindset, encouraging a focus on other health changes when experiencing no weight loss, a constant reminder of how to follow the dietary approach, and help with developing strategies to overcome barriers. Participants valued the rapport with the dietitian and the face-to-face interaction (either in-person or via Zoom). Participants reported that the research dietitian helped to build knowledge of how to follow their allocated dietary intervention, which was particularly important for the CER intervention compared to the fasting interventions for providing advice around dietary choices. Whereas for participants who perceived there was lack of contact from the research dietitian, participant engagement was negatively impacted and was reported as one of the reasons for lost-to-follow up.

#### 2.2 Accountability

Participants often mentioned that the need to report back to someone, in particular the research dietitian, regarding their progress kept them accountable and helped with continued engagement across all dietary interventions. Being a part of a study, rather than undertaking their dietary intervention solo, also made participants feel more accountable.

#### 2.3 Self-monitoring

Self-monitoring, particularly through self-weighing, was generally seen as helpful for engagement, particularly when experiencing weight-loss. However, self-monitoring was sometimes identified as a barrier when experiencing limited weight-loss (i.e., weight gain, or perceived slow or no weight loss), often demotivating participants to follow their dietary intervention and questioning its effectiveness. The LADs revealed that participants often fluctuated between states of experiencing weight loss and no weight loss, which negatively impacted their motivation.

*I guess the first week of the fortnight I was feeling great…I'd lost weight. I was feeling on top of the world.* (LAD from participant 1084; Female; IF:2D)

*…just a few days ago, I weighed in again, and hadn't lost any weight, had lost 100 grams…kind of just made me go well, "Stuff it all".* (LAD from participant 1084; Female; IF:2D)

### Theme 3: An individualised approach is sometimes needed

Engagement to the dietary interventions was sometimes influenced by whether the dietary approach they were assigned to was perceived to match participant needs. Subthemes 3.1 and 3.2 related to the CER intervention, while 3.3 relates to the IF interventions.

#### 3.1 Increased nutrition knowledge

Where nutrition knowledge was perceived to be lacking, a focus on increasing this knowledge helped with following the CER intervention. Perceived knowledge gained included increased awareness of the fat content of certain foods, suggested brands with a healthier nutrient profile, understanding how certain foods can affect energy levels, etc.

#### 3.2 Focus on regular eating

A focus on ‘regular eating’ (i.e., aiming for three meals over a 24-h period and consuming snacks in between meals when hungry) was identified by participants as important for following the CER intervention. This was important for participants that described their pre-baseline eating pattern as irregular, such as snacking frequently and/or having long periods of limited intake. Dietetic advice was important for developing participants capability to regulate their eating patterns to reduce becoming overly hungry and then make poor food choices.

#### 3.3 Limited focus on non-fasting periods

Some participants following the IF interventions, particularly those allocated to the IF:2N intervention, reported that overconsuming or making poor food choices on non-fasting days or nights hampered weight loss and motivation, and suggested that strategies to assist with this were needed. Other participants reported using strategies to reduce overeating on non-fasting days that did help with their non-fasting days or nights, which included keeping busy, mindful eating, and planning healthy food options for the days directly after fasting.

*Something else I've been doing is I'm prepping—that I never used to do. I'm prepping healthy snacks for work, whether they'd be—and these are including the days where I'm not fasting. I might cut up some cucumber and capsicum and just something easy like that and put it in a container and take it to work for me to snack on.* (LAD from participant 1062; Male; IF:2N)

### Theme 4: Personal motivation and attitudes

This main theme relates to the intrapersonal level of the SEM, in particular personal motivation and attitudes identified to influence engagement across the interventions.

#### 4.1 Motivation

Participant’s motivation fluctuated throughout the 24-week intervention period. Being highly motivated or having multiple motivations positively influenced participant’s engagement. Motivation came from various sources such as the desire to lose weight, improved health, altruism (i.e., helping fellow shift workers), or being a family role model. Conversely, a lack of motivation was identified as a negative influence on engagement. Lack of motivation was described as stemming from not experiencing weight loss, experiencing fasting side effects, less frequent catch-ups with the dietitian, influence of social occasions/outings, fatigue, mental health, and sickness.

*I’m really struggling to…find…motivation again…I’m super sleepy all the time.* (LAD from participant 1044; Female; IF:2D)

#### 4.2 Positive mindset

A positive mindset supported participants to work through issues they were experiencing. This mindset was sometimes reported as an existing part of their personality or influenced by past dieting experiences, but often was influenced by dietetic support and external social support. Focusing on non-weight outcomes*,* such as freedom of movement, feeling energetic, or just a general ‘healthy’ feeling, helped with a positive mindset, which promoted their continued engagement with their allocated intervention. This focus sometimes overrode the negative feelings associated with not seeing weight-loss.

#### 4.3 Lack of time

A perceived lack of time was a personal attitude that negatively influenced engaging with the dietary intervention, especially for the CER intervention. However, some participants were able to work on this with the support of the dietitian and be organised with their diet plan.

### Theme 5: Physiological influences of eating behaviours

This main theme relates again to the intrapersonal level of the SEM, in particular perceived enablers or barriers of physiological influences of eating behaviours across the interventions.

#### 5.1 Fatigue

General life activities and/or working night shift was often attributed to fatigue and was described as a barrier to following the dietary interventions for many participants, particularly by those following the CER intervention. Fasting was particularly challenging when it coincided with two consecutive night shifts, as this is a period of limited sleep and high fatigue. Fatigue often led to reduced motivation to follow the dietary intervention and increased craving foods high in sugar or fat, and triggered thoughts of needing to eat or to drink caffeinated beverages as a way of boosting energy.

*Just with the lack of sleep, I tend to fall into too many coffees on night shift and obviously go over my calories with the coffee.* (Interview with participant 1041; Female; CER)

#### 5.2 Emotional eating

Emotional eating encompassed discussion of eating to suppress or soothe negative emotions (e.g., stress, anger, fear, boredom, sadness, loneliness, poor mental health, etc.) and was identified as a barrier for following the dietary strategies.

#### 5.3 Keeping busy

To help with the physiological influences associated with eating, participants frequently described trying to keep busy to distract themselves from hunger or boredom eating, such as keeping busy at work, going for a walk, housework, cooking, sleeping, gardening, etc.

#### 5.4 Mindful eating practices

Mindful eating practices such as keeping free from distractions, focusing on the eating experience, slowing down eating, and tuning into hunger and fullness cues, were reported to help with addressing hunger or emotional eating for a small number of participants. While the dietary interventions did not aim to incorporate this approach, this was something that participants had learned in the past or had naturally progressed out of following the fasting interventions.

### Theme 6: Social support at home and work

This main theme relates to the interpersonal level of the SEM, with social support and the influence of eating with others particularly important at this level across the interventions.

#### 6.1 Supportive family, friends or colleagues

Family support included joining in on the dietary changes to make it easier and/or providing assistance (e.g., preparing permitted food items for fasting, or help with cooking for the CER intervention). Support from friends or colleagues also came from encouraging comments related to health changes, or encouragement to continue with the strategy and refraining from offering unhealthier food options.

#### 6.2 What others are eating

If family, friends or colleagues did not partake in making the same dietary changes, what others were eating sometimes made it difficult for participants to follow their dietary intervention. This was particularly the case for the fasting interventions, as it was more difficult for family members or colleagues to join in on the dietary changes as opposed to the CER intervention. Social occasions or outings were particularly challenging for the IF:2D intervention, as participants found it difficult to plan their fasting to incorporate these occasions and felt a sense of ‘missing out’.

*If I had things planned for those* [fasting] *days, like if I was going to a party or someone's birthday, I couldn't eat anything outside of what I was programmed to eat, what I had scheduled to eat. So, from a social aspect, I felt like I was missing out in that sense.* (Interview with participant 1055; Male; IF:2D)

### Theme 7: Work structure and environment

This main theme related to the organisational/community levels of the SEM, with organisational practices and the workplace/community environment as identified as important influences for engagement across the interventions.

#### 7.1 Busy work

A busy work schedule helped participants take their mind off eating and minimised boredom eating. However, if busy work led to interrupted meal breaks this negatively influenced following the dietary intervention, particularly for the CER and IF:2N interventions.

#### 7.2 Regular work breaks

Structured work breaks or the ability to take breaks ad hoc, was identified as important for regular eating patterns to prevent becoming overly hungry. This was important for distributing the provided food items over the work shift period for the fasting interventions and for regular food intake for the CER intervention.

#### 7.3 Shift schedule

A nightshift work schedule, long work hours and the disruption this causes to general life and sleep patterns, was identified as a barrier subtheme to following the dietary interventions due to the ‘fatigue’ and ‘lack of time’ barriers discussed previously.

#### 7.4 Availability of unhealthy food

Unhealthy food available in the workplace and wider community was identified as a barrier subtheme. This food availability was attributed to available outlets such as vending machines, take away stores, or via existing work practices (i.e., group purchasing of take-away, communal meals, colleagues sharing unhealthy snacks). While some participants found that they could avoid this temptation because of their motivation to follow their dietary intervention and *“didn’t cave”,* for others it was difficult to resist.

*I've had a few days at my day job…and there's just always so much junk food there. I just ate like crazy.* (LAD from participant 2062; Female; IF:2N)

#### 7.5 COVID-19 stressors

A large period of the SWIFt study was during the COVID-19 pandemic. For some participants, this experience negatively influenced their mental health and stress levels, both at home and in the workplace, and lead to using food as a way of alleviating stress. Many of the professions recruited for the study were significantly affected by the pandemic such as nurses and paramedics, due to increased workload and changes in work practices.

## Discussion

This study provides a unique exploration of the experiences of night-shift workers following three different dietary weight-loss interventions, to provide an understanding of how intervention features and external enablers/barriers influence participant engagement. In terms of aspects of the interventions that helped with engagement, simplicity, support, and accountability were key and were linked predominantly with motivation and capability behaviour change domains. However, for some participants, strategies for managing food intake on non-fast days and the option to choose to fast during a day shift/day off or night shift were needed for the fasting interventions, and for some participants a more structured approach was needed for the CER intervention. A number of barriers and enablers external to the interventions were also identified that influenced engagement ranging from the individual level (e.g., limited time and fatigue), social level (e.g., family and colleague support), and organisational/community (e.g., regular work breaks and access to healthy food).

### Simplicity: an important aspect for the SWIFt interventions but flexibility is needed

The IF interventions adopted in SWIFt were perceived to provide a straight forward and flexible approach (subtheme 1.2) to weight-loss for most participants, via a clear prescription for only two days per week aided by the provision of food for these days. Previous studies have also reported that the twice per week IF regimen is perceived as a less complicated approach compared to CER and possibly easier to adhere to [42, 43], but there is limited supporting evidence and none published for shift working populations. A qualitative study with women at risk of breast cancer who trialled a twice per week IF approach after previous CER attempts, found that participants perceived the approach to be more manageable and less cognitively demanding due to the focus on only two days per week and the prescriptive rules on fasting days [44]. However, for some SWIFt participants, the flexibility of the IF interventions was limited due to their shift work schedule (subtheme 1.3). Greater flexibility in the choice of fasting days (day or night) may enhance engagement and resulting weight-loss outcomes and should be considered for night shift workers in future scale-up of the interventions.

While the prescription of fasting aided engagement, it was identified that for some, strategies were needed to help manage food intake on non-fasting days or nights (subtheme 3.3). Some participants had the knowledge and capability to do this, while others described lack of guidance on these days as a challenge that negatively affected their engagement. The evidence on how to counsel people on non-fast days when following twice per week IF is mixed, with a recent systematic review of IF interventions showing no clear pattern between dietary advice on non-fast days, engagement or weight-loss outcomes [19]. Future research is needed to determine whether dietary advice on non-fast days influences engagement and weight loss outcomes. For adoption of the SWIFt IF interventions, providing some general dietary advice for non-fasting days should be considered to potentially enhance engagement, while maintaining the ease of the approach.

There were aspects of the CER approach that were also perceived as providing simplicity for participants, with a focus on small changes to their existing diet that often aligned with family life (sub-theme 1.6). However, for some participants, more structure was needed to this approach, such as a detailed and tailored meal plan for each day of the week, rather than suggested modifications to their current diet.

The findings of this study indicate that the incorporation of provided foods in the design of SWIFt addressed some of the known barriers to healthy eating during night shift [11] and the provided foods were also perceived to add to the ease of following the dietary interventions, especially for the fasting interventions (subtheme 1.1). The provision of portion-controlled, healthier foods has been identified as a strategy that often increases adherence to a weight-loss intervention, however this has not been explored in twice per week IF diets [43]. In the context of night shift work, Sooriyaarachchi et al. [17] explored the experiences of participants following a dietary weight-loss intervention using a provided low-calorie/energy meal replacement and also found the meal replacement to be simple and convenient making it feasible even during busy night shifts [17]. Practical and innovative ways of making portion controlled, healthier meals and snacks available for night shift workers are needed, such as workplace provision, providing accessibility (e.g., healthy vending machines, changes to on-site vending arrangements), or providing guidance for workers on what to prepare/purchase with appropriate facilities to store and prepare foods. For example, a study that implemented healthier options in hospital vending machines showed a reduction in daily energy intake for employees, without changes in vending revenue [45].

### Support and accountability: important aspects of the SWIFt interventions for motivation

Dietetic support and associated accountability were identified as positive intervention components across all SWIFt dietary interventions contributing to motivation and ongoing engagement (subthemes 2.1 and 2.2), similar to what has been reported in the literature for the general population [46, 47]. A systematic review of qualitative studies that document the strategies used in weight loss attempts, identified that professional support (i.e., personal trainers, doctors and nutritionists) was important for accountability and motivation [47]. Contact with a dietitian has also been found to be associated with increased effectiveness of weight management interventions [46]. It is acknowledged that dietetic support may be a cost and resource dependent element of a dietary intervention. The time and funding needed to implement health initiatives is often cited as a barrier for workplaces [48] and the cost to the individual may be a barrier for seeking support [49]. Innovative ways to fund this support are required that also retain the important behaviour change components. Possible avenues for exploration include the dietetic support available through government funding for chronic disease management [50]. For example, patients in Australia with a chronic health condition are entitled to five government subsidised allied health consultations [50]. Some workplaces already offer nutrition health promotion initiatives [50] and the findings from this study can be used to tailor these initiatives to make them more specific to night shift workers. Other benefits to an organisation that accompany improved employee health outcomes such as decreased absenteeism, increased job satisfaction, and increased productivity should be a factor to consider in the decision to fund such initiatives [51].

Most SWIFt participants found that self-monitoring their weight helped with their motivation to follow their dietary goals, however, there were mixed views as to whether monitoring weight was helpful when not experiencing weight loss (subtheme 2.3). Self-monitoring behaviours (e.g., dietary change) and outcomes (e.g., weight change) is a well-documented technique reported in the behaviour-change literature [47, 52–54]. A review undertaken by Hartmann‐Boyce et al*.* [47] identified that some participants reported monitoring weight led to feelings of self-efficacy/self-control and increased accountability for one’s own actions, whereas others experienced shame and fear related to this practice [47]. Similarly, Deslippe et al*.* [55] reviewed barriers and facilitators to diet, physical activity and lifestyle behaviour intervention adherence, and found that while weight loss helped reinforce commitment to an intervention, failing to see these changes or a ‘focus on weight’ often led to dissatisfaction in progress, hindering behaviour maintenance. Evidence is lacking as to what may determine this individual variability of the relationship between self-monitoring of weight, adherence and weight change [56]. Participants in the SWIFt study stated that receiving dietetic support was beneficial in shifting their attention away from weight-related concerns. In a future scale-up of the SWIFt dietary interventions, this aspect could be strengthened by: increasing participants understanding of valuable health changes in addition to weight [55], having more focus on these health changes (e.g., increased energy, reduced fatigue, reduced blood pressure, etc.), and monitoring these aspects (e.g., rating energy levels throughout the day/night). Providing a focus on health changes important for night shift workers, such as fatigue, may be especially beneficial.

### Barriers and enablers external to the SWIFt dietary interventions

Several external enablers and barriers to the SWIFt dietary interventions were identified that offer the opportunity to further enhance scale-up of the dietary interventions. A multi-component approach can address the complex interaction of individual, social and environmental influences of weight management as evident through the lens of the social-ecological model (SEM) [57] and workplaces have been identified as a setting that can incorporate strategies across these levels [51, 58, 59]. Social support from colleagues was identified as an enabler for the SWIFt dietary interventions at the interpersonal level (subtheme 6.1), and workplace health interventions are one way to harness this enabler by encouraging multiple employees to undertake an intervention simultaneously and harnessing existing peer support [60]. A workplace healthy lifestyle intervention for night shift workers that has incorporated a team-oriented format, found that there was significantly less weight gain compared to a control group [61].

In line with previous research, fatigue was often reported as negatively impacting engagement in the SWIFt interventions (subtheme 5.1) [11, 16, 62]. The uniqueness of this barrier to night shift workers is evident when looking at the self-perceived barriers of weight loss in the general population, with fatigue not identified as a common barrier [63]. Incorporation of fatigue management strategies, such as sleep hygiene recommendations or behavioural therapy to improve sleep [64–66] alongside a dietary weight-loss approach could be trialled to address this barrier. Furthermore, continued work on optimising shift scheduling to reduce fatigue could further enhance future scale-up [57]. While there is limited research on the optimal shift schedule, current guidelines recommend that schedules include: ≤ 3 consecutive night shifts; shift intervals ≥ 11 h; and ≤ 9 h shift duration to reduce fatigue [67]. High quality research is currently being undertaken to help support these recommendations, such as a trial to test the effectiveness of appropriate shift intervals [68]. Consideration of adjunct fatigue management strategies is recommended for any future scale-up of the SWIFt interventions.

### Strengths and limitations

This study is the first to explore the experiences of night shift workers while following three different weight-loss interventions and provides insight for future weight loss approaches. Night-shift working participants were from a broad range of demographics (e.g., occupation, gender, age, and shift type) and type of dietary intervention, which provides a rich data set to explore the influencing factors for engaging with the study. However, it is acknowledged that a higher percentage of participants that identified as being from a European or Australian cultural and ethnic background chose to participate compared with participants who declined to participate, which may limit the generalisability of findings. The longitudinal data collection and opportunity to build rapport between researcher and participant also added to the richness of data collected by allowing for exploration of experiences over time [28]. A limitation to the study is that interviews and LADs were optional, so participants involved may differ in their experience (e.g., may be highly motivated). However, a range of barriers were identified which reflects the strength of the maximum-variation sampling and the inclusion of participants who chose to withdraw from the study (*n* = 2) or were lost to follow-up (*n* = 3). This qualitative study is based on participants who agreed to be a part of a weight-loss study, so the findings cannot be generalised to all night-shift workers. Further investigation is warranted around the factors that influence night-shift workers to take part (or not) in a dietary weight-loss study to optimise uptake of an intervention. Lastly, eligibility for the wider SWIFt study was determined by a participant’s BMI, however future shift work studies should have broader inclusion criteria that are not primarily focused on BMI in line with recent guidelines [69]. While this study aimed to explore the experiences of night shift workers, further publications aim to evaluate the success of the intervention for main outcome measures (e.g., weight loss and insulin resistance).

## Conclusions

The findings of this qualitative study found that the straight forward format of the IF interventions and the small changes to participant’s existing diet for the CER intervention, in combination with the provision of portion-controlled meals/snacks for all interventions, were key components for engagement. In addition, across all interventions the support and accountability provided by the research dietitian and the requirement for self-monitoring, were important and were linked predominantly with motivation and capability behaviour change domains. Participant’s perspectives suggest that modifications to increase engagement include: option to choose between day or night fasting and inclusion of strategies for non-fasting days for the IF interventions, and across all interventions, addition of fatigue management initiatives and an increased focus on non-weight related health changes during periods of slowed weight loss. In addition, across all interventions, engagement may be enhanced by harnessing the enabling effect of social support through implementation in workplace settings and addressing organisational barriers, such as providing a healthier food environment.

## Supplementary Information

Supplementary Material 1.

Supplementary Material 2.

## Data Availability

No datasets were generated or analysed during the current study.

## Abbreviations

IF: Intermittent fasting. This study focuses on a form of intermittent fasting known as twice per week fasting (often referred to as the 5:2 diet) which involves habitual dietary intake five days per week) and modified ‘fasting’ days two days per week, with ‘fasting’ days (or low energy days) prescribed as a significantly reduced daily energy intake of approximately 2100 – 2500kJ (500 – 600 calories)
IF:2N: Intermittent fasting with two fasting days aligning with a night shift
IF:2D: Intermittent fasting with two fasting days during a day shift or day off
BCT: Behaviour change taxonomy
CER: Continuous energy restriction
COM-B: Capability (C), opportunity (O), motivation (M) behaviour change (B) model
SEM: Social ecological model
SWIFt: Shift Weight using Intermittent Fasting in night shift workers study
TDF: Theoretical domains framework
