# Supplementary material for: The experiences of night shift workers following three different dietary weight loss interventions: a qualitative study using behaviour change theory
Source: Int J Behav Nutr Phys Act. 2025 May 28;22:64. doi: 10.1186/s12966-025-01750-7 (PMC12117937; doi:10.1186/s12966-025-01750-7)
Supplement: Supplementary file 2 — Supplementary Material 2. [file 12966_2025_1750_MOESM2_ESM.docx]

### Additional file 2

### Longitudinal audio diary (LAD) guide

Thank you for agreeing to participate in this study. Please send us at least 1 audio-diary entry per fortnight (for the next 24 weeks) based on your experience in following your allocated SWIFt diet. We are especially interested in what has worked or not worked well for you over each 2-week period and what you think helped or did not help with your experiences. We encourage you to record a diary entry as soon as practical after an experience (e.g. on the day the experience occurred). However, if this is not practical, you may provide a fortnightly overview of your experience(s). If you have had multiple experiences during the 2 weeks, please share with us your most memorable positive experience and your most memorable negative experience. For each diary entry, please state the **date** and **time** of your entry.

We would like to hear a brief overview of your experience as part of your diary entry in order to understand what it is like to follow the diet. We provide some prompts below to help you think about your diary entries, but please feel free to discuss anything that you feel is relevant to your experience of the diet.

1. On one or more occasions, did you experience difficulty with following the diet?
2. On one or more occasions, did you experience times when following the diet was easy?

For each experience, could you talk about:

- What was happening?
- Was there anyone else involved?
- What were you thinking at the time? What did you feel at the time?
- What did you do? Were you aware of any reasoning behind what you did?
- Do you think that the diet design (e.g. the way that the diet required changes to your behaviour) influenced this experience? If so, how?
- Do you think that the SWIFt study processes and resources (e.g. dietitian consultations, food provided, etc.) influenced this experience? If so, how?

1. Could you provide some more general comments about your experience of the diet?

- What factors (if any) do you think helped/hindered you in following your diet strategy?
- How are you feeling about your progress?
- Any other comments you want to make about following your diet strategy the past 2 weeks?

To assist with completing audio diaries, the research team will regularly remind you of the audio diary via text message or email (whichever you prefer). The following tips may assist with planning your audio diaries:

- In order to capture your experiences, thoughts, and feelings as they happen throughout your participation, feel free to pick up your phone and record an entry whenever you are reflecting on your experiences of the diet and you have time
- If you have an experience that you would like to share, try to complete your audio diary ASAP
- Try to pick a regular day and time each fortnight to complete an audio diary entry
- Put a reminder in your phone for the day/time for your audio entry

Ideally, record your voice within a quiet environment to maximise audio quality
